# Cancer Dormancy: A Regulatory Role for Endogenous Immunity in Establishing and Maintaining the Tumor Dormant State

**DOI:** 10.3390/vaccines3030597

**Published:** 2015-07-30

**Authors:** Constantin N. Baxevanis, Sonia A. Perez

**Affiliations:** Cancer Immunology and Immunotherapy Center, Saint Savas Cancer Hospital, 171 Alexandras avenue, Athens 11522, Greece; E-Mail: perez@ciic.gr

**Keywords:** targeted therapies, tumor-oriented therapies, tumor microenvironment, tumor dormancy, endogenous immunity, immunoediting, immunotherapy, cancer stem cells

## Abstract

The significant contribution of host immunity in early tumorigenesis has been recently recognized as a result of our better understanding of the molecular pathways regulating tumor cell biology and tumor-lymphocyte interactions. Emerging evidence suggests that disseminated dormant tumor cells derived from primary tumors before or after immune surveillance, are responsible for subsequent metastases. Recent trends from the field of onco-immunology suggest that efficiently stimulating endogenous anticancer immunity is a prerequisite for the successful outcome of conventional cancer therapies. Harnessing the immune system to achieve clinical efficacy is realistic in the context of conventional therapies resulting in immunogenic cell death and/or immunostimulatory side effects. Targeted therapies designed to target oncogenic pathways in tumor cells can also positively regulate the endogenous immune response and tumor microenvironment. Identification of T cell inhibitory signals has prompted the development of immune checkpoint inhibitors, which specifically hinder immune effector inhibition, reinvigorating and potentially expanding the preexisting anticancer immune response. This anticancer immunity can be amplified in the setting of immunotherapies, mostly in the form of vaccines, which boost naturally occurring T cell clones specifically recognizing tumor antigens. Thus, a promising anticancer therapy will aim to activate patients’ naturally occurring anticancer immunity either to eliminate residual tumor cells or to prolong dormancy in disseminated tumor cells. Such an endogenous anticancer immunity plays a significant role for controlling the balance between dormant tumor cells and tumor escape, and restraining metastases. In this review, we mean to suggest that anticancer therapies aiming to stimulate the endogenous antitumor responses provide the concept of the therapeutic management of cancer.

## 1. Introduction

The endogenous immunological response during the natural course of cancer constitutes the concept of cancer immunomodulation as described in the “immunoediting” hypothesis almost 1.5 decades ago [1]. The immunoediting process is based on the knowledge obtained from progresses in our understanding of mechanisms regulating tumor cell immune recognition and immune evasion. During immunoediting, elements of the innate and adaptive immune system initially eliminate immunogenic tumor cells (elimination phase). Then comes a rather long period during which the immune system continuously interacts with the tumor establishing a dynamic state of equilibrium which keeps the tumor cells in a dormant state. Thus, tumor immune surveillance is a major component of long, or even permanently, lasting tumor dormancy, however, only when tumor cells are immunogenic [2]. The equilibrium phase will progressively fade in the presence of epigenetic alterations, significantly affecting the biology of tumor cells, making them less immunogenic, highly suppressive, with a high angiogenic output. This may seriously impact the balance between effector and regulatory cell compartments by favoring the infiltration and accumulation of regulatory T cells (Tregs) and myeloid-derived suppressor cells (MDSC) within tumors. In this way, effector T cells that do infiltrate the tumor will be negatively controlled by these regulatory cellular subsets and inhibitory molecules. The outcome of this dysregulated balance between effector and regulatory cells is critical for the tumor to escape immune control [3]. The immunoediting hypothesis provided an immune-mediated control of tumorigenesis by postulating opposing host-protective and tumor-promoting functions of the immune system. Based on this theory, studies, later on, confirmed the role of endogenous adaptive antitumor immunity both as prognostic and predictive biomarkers [4,5]. To this end it was demonstrated that the immune contexture (presence, location, and density of T cells and cytokines within tumors) is related with a favorable prognosis, hence emphasizing the ability of the immune response to maintain a subclinical tumor in an equilibrium state [5]. The major role of the endogenous intratumoral immune reaction could improve our understanding of tumor evolution and have important consequences in clinical cure of cancer.

## 2. Modulation of the Endogenous Antitumor Immunity

Immune-based therapies delivered through active immunotherapy, include vaccines, which are used to activate pre-existing host antitumor immune cells but to also induce new ones to react against the tumor and induce tumor cell destruction. Active immunotherapy in humans has resulted in potent immunological and occasionally clinical responses and regression, mostly in cases when responses against multiple tumor epitopes could be readily induced [6].

The ideal system would entail vaccination strategies that can generate robust antitumor immunological responses with durable tumor-specific memory, while boosting and/or inducing an active endogenous response to reinforce the tumor eradication. Cancer immunoediting enabled the promotion of new modalities in cancer immunotherapy for improving antitumor immunity. Thus, great effort has been put forth to design more effective active, antigen-specific immunotherapies by increasing the number and the quality of tumor-specific cytotoxic T lymphocytes (CTLs), revealing additional immunogenic tumor peptides and reversing tumor-induced suppression [7,8]. To this end, various forms of vaccination strategies also in combination with other therapeutic modalities have been explored to elicit robust immune responses to tumor antigens (Table 1). Under ideal circumstances, the endogenously induced anti-tumor T cells could migrate to the tumor site and directly lyse tumor cells, while rescuing endogenous immune cells from the tumor-induced immunosuppression [9,10,11]. However, the tumor environment is usually so immunosuppressive that it is difficult to appropriately release these brake mechanisms on antitumor responses.

**Table 1:** Phase II and III clinical trials, utilizing various therapeutic vaccine platforms.

Study Name	Targeted Antigen	Vaccine	Cancer Type	Refs.
IMPACT	PAP	Mo/DCs PAP-GM-CSF	mCRPC	[12]
PROSPECT	PSA	rV-PSA-TRICOM rF-PSA-TRICOM	mCRPC	[13]
PANVAC	MUC1/CEA	DCs rV-PSA-TRICOM rF-PSA-TRICOM	Colorectal	[14]
GVAX	Multiple on allogeneic tumor lines	T47D and SKBR3 lines secreting GM-CSF	Breast	[15]
DERMA	MAGE-A3	Recombinant protein	Melanoma	[16]
MAGRIT	MAGE-A3	Recombinant protein	NSCLC	[17]
IMPRINT	Mixture of 11 naturally presented renal Ca tumor peptides	soluble	Renal	[18]
BIOVAXID	Id-IgG on B-cell lymphoma	Id-IgG-KLH	Follicular lymphoma	[19]
GV1001	Telomerase	Soluble 16-mer	Melanoma	[20]
TG4010	MUC1	MVA MUC-1-IL-2	Prostate NSCLC	[21,22,23]
CDX110	EGFRvIII	Soluble 13-mer	Glioblastoma	[24]
Stimuvax	MUC1	25-mer MUC1 liposome (BLP25)	NSCLC	[25]

PAP: prostatic acid phosphatase; PSA: Prostate specific antigen; MUC1: Mucin 1; CEA: carcinoembryonic antigen; Id: Idiotype; IgG: Immunoglobulin G; EGFRvIII: Epidermal growth factor receptor variant III; Mo/DCs: monocytes/dendritic cells; GM-CSF: granulocyte macrophage-colony stimulating factor; rV: recombinant vaccinia virus; rF: recombinant fowlpox virus; TRICOM: triad costimulatory molecules; KLH: keyhole limpet haemocyanin; MVA: modified vaccinia ankara virus; IL-2: Interleukin 2; mCRPC: metastatic castrate resistant prostate cancer; NSCLC: non small cell lung carcinoma.

Immunomodulatory monoclonal antibodies, acting either as agonists targeting immunoenhancing receptors, or as antagonists directed at co-inhibitory receptors, have been demonstrated to enhance the endogenous antitumor immunity [26]. Combining vaccines with such immunostimulatory monoclonal antibodies is a quite promising field of immune vaccine combination therapy based on a multifaceted activation of patients’ immune system, accomplished through the enhancement of endogenous antitumor immune responses, and induction and maintenance of vaccine and tumor-specific immunity [27,28]. Improvements have also been made in the treatment of cancer through the development of “targeted” therapies, those that specifically inhibit dysregulated oncogenic pathways. In addition to their direct antitumor effects, these agents could facilitate recognition and sensitivity to effector functions by cytolytic lymphocytes of the innate and adaptive immune system, thereby sensitizing cancer cells to immunotherapy [8,29,30,31,32]. A major goal, therefore, is to develop rational combinatorial approaches that merge the significant benefits of oncogenic pathway disruption using targeted agents, with the unique ability of immunotherapy to mediate long-term responses in certain cancers. Such therapeutic approaches used in combination or sequentially will be required to harness the full potential of the endogenous antitumor immune response.

## 3. Tumor Dormancy as a Result of Endogenous Immune Surveillance

The functional dichotomy of the immune system to recognize and eliminate, but also to shape the immunogenicity of tumor cells, enabled a better understanding of the relationship between the endogenous antitumor immunity and cancer, and also provided insights into the management of malignant diseases. In recent years, the tumor immune microenvironment has attracted attention, as this microenvironment plays a pivotal role in regulating tumor development and/or progression. Tumor infiltrating lymphocytes (TIL), including several different lymphocyte subsets, generally possess a critical role in the positive and negative regulation of antitumor immunity. Indeed, the pattern of immune cell infiltrates emerged as a key criterion for predicting disease-free survival and overall survival [33]. Data from human colon cancer, but also other types of malignancy, have convincingly shown that intratumoral immune signatures constitute an independent significant prognostic factor, which is superior compared to the standard TNM classification [34]. There are several reports to demonstrate an association between infiltration of immune cells with a favorable prognosis. For example, high densities of memory CD8+ T lymphocytes were associated with improved clinical responses [5,35]. More importantly, the presence of TIL has been also shown to correlate with increased survival in the patients who had complete responses after chemotherapy [4,5] or neoadjuvant chemotherapy [36,37] providing evidence that immune cell infiltration represents not only a favorable prognostic factor, but also could be predictive for the outcome of conventional chemotherapies. On the other hand, there are also studies to show that the number of cells exerting suppressor function (*i.e.*, Tregs and MDSCs) are also increased and migrate to the tumor sites, impairing the ability of host’s immune system to defend against tumor progression [6,9]. Moreover, the tumor microenvironment is rich in cytokines and other inflammatory mediators, which positively or negatively regulate TIL-mediated immunosurveillance [1]. For instance, TGF-β, IL-10 and IL-17 have been demonstrated to initiate immunosuppressive networks whereas Th1-associated IFN-γ stimulates tumor-specific immunity [10,11,26,27,28,29,30].

Presently, it is not known as to which extent the immune composition of human tumors may influence the immunoediting process. Adaptive immune signatures intratumorally may reflect the fact that host antitumor immunological responses may protect the host from rapid tumor growth and thus prolong overall survival. As also mentioned above, immune signatures may be predictive of subsequent conventional therapies: it has been suggested that tumor antigens released through the destruction of tumor cells by chemotherapeutic agents can trigger efficient antitumor immunity, particularly in those patients whose immune system has been primed against tumor antigens before chemotherapy [38]. Thus, conventional therapies for cancer may profit from the participation of the immune system and *vice versa*. Immunogenic tumor-cell death and stimulation of the immune system through transient lymphodepletion, which subverts immunosuppressive mechanisms, belong to the contribution of conventional therapies, whereas immunotherapies contribute through sensitization of the tumor against subsequent chemotherapeutic treatments [39]. Clinical evidence indicates that the enhancement of endogenous immune response against tumor cells via standard treatments such as radio, chemo and hormonal therapies or targeted therapies is associated with improved clinical outcome in patients with various types of cancer [40,41,42,43] (Table 2). The discovery of immune checkpoint inhibitors and their implementation in clinical trials represents a modality of paramount importance for the development of robust anticancer immunosurveillance. Immune checkpoint therapies through the use of immunostimulatory monoclonal antibodies have revolutionized the field of cancer immunotherapy inducing durable clinical responses in a substantial number of patients with various types of cancer [44]. The clinical success of immune checkpoint antibodies against CTLA-4, PD-1 and PD-L1 is based on the fact that the regulatory pathways they target result in the enhancement of endogenous antitumor immune responses. Recent published data on whole exome sequencing of tumor tissue from human non-small lung cancer and melanoma has revealed a strong association of the clinical response to checkpoint inhibition with a particularly high mutational load [45]. The findings from these studies improved our understanding regarding the nature of the antigens that allow the immune system to specifically recognize malignant cells. Emerging evidence suggests that the immune response to patient-specific neoantigens that arise as a consequence of tumor-specific mutations comprises a substantial part of the endogenous anticancer activity of immune checkpoint immunotherapies [46]. Thus, boosting neoantigen-specific T cell responses is promising for further improving clinical efficacy of immunotherapies as a result of enhanced endogenous antitumor immunity and robust tumor immunosurveillance (elimination). 

The cancer immunoediting theory postulates that a tumor is “edited”. However, tumors are not always edited. Certain types of cancer may never be detected clinically, because their establishment is limited via the action of adaptive immune responses. In such a case, we may propose that the elimination phase is complete. Actually, this has been documented both preclinically and clinically in immunocompromised animals, and humans as well, who were demonstrated to have high rates of spontaneously arising or induced cancers [47]. Nevertheless, tumor editing implies that the elimination phase is incomplete, resulting in a state of equilibrium between the developing tumor and the immune system, which may span for years. We may envisage that during this period tumor cells will resist the selective pressure expressed by the immune system, by acquiring genetic and epigenetic changes that allow their progression, despite an ongoing immune response [48,49]. For example, tumors from patients with advanced disease were found to have deficiencies in their MHC I processing pathway, being unable to present tumor peptides in the context of MHC class I alleles to CD8+ CTLs [50,51], while other tumors have down regulated the expression of peptide sequences that could serve as antigens for T cells [52]. A direct involvement of MHC class I expression in immunomediated dormancy was recently demonstrated in a novel mouse tumor model of permanent immunomediated metastatic dormancy [53]. In this model, spontaneous metastases derived from an H-2 class I negative aggressive fibrosarcoma clone were kept at permanent dormancy via immunomediated and oncogenic suppression mechanisms. The primary local tumor generated from this clone was H-2 class I positive, recovering the surface expression of the three H-2 molecules. Immunodepletion of immunocompetent hosts’ T or NK cells awoke the dormant disseminated metastatic cells, which then metastasized in the lungs of the mice; these metastases were all H-2 class I positive. The results (i) suggest that the expression of MHC-I molecules on these metastatic cells may prolong the dormant state via immunomediated mechanisms and (ii) promote that treatments activating CTLs or rescuing CTLs from tumor-mediated immunosuppression or even by activating NK cells. To this end, it is worth mentioning that MHC class I defects, besides enabling tumor immune evasion, also directly promote cancer progression, growth and oncogenicity, suggesting a new role of MHC molecules as tumor suppressor genes [54,55]. Therefore, MHC class I expression of a tumor should be considered as a biomarker predicting its clinical progression implying that quantitation of MHC class I expression in tumor biopsies or surgically excised tumors should be accurately performed before any treatment.

**Table 2:** Potentiation of the endogenous antitumor immunity with conventional cancer therapies.

Treatment	Mechanisms for Synergistic Effects
Chemotherapy	“Immunogenic” cell death Alterations in tumor cell phenotype Inhibition of regulatory cells Homing to tumors
Radiation	Increased tumor antigen presentation Release of pro-inflammatory cytokines Promotion of tumor antigen cross-presentation
Kinase inhibitors	Promotion of DC maturation T cell priming, differentiation in memory cells Sensitization of tumor cells to immune-mediated killing Impairment of immunosuppression
Hormonal therapy	Increase of T cell levels in lymphoid tissues T cell sensitivity to antigen-specific stimulation T cell infiltration into the prostate

## 4. Endogenous Immunity and Tumor Cells as Major Players for Tumor Dormancy

**Figure 1:**
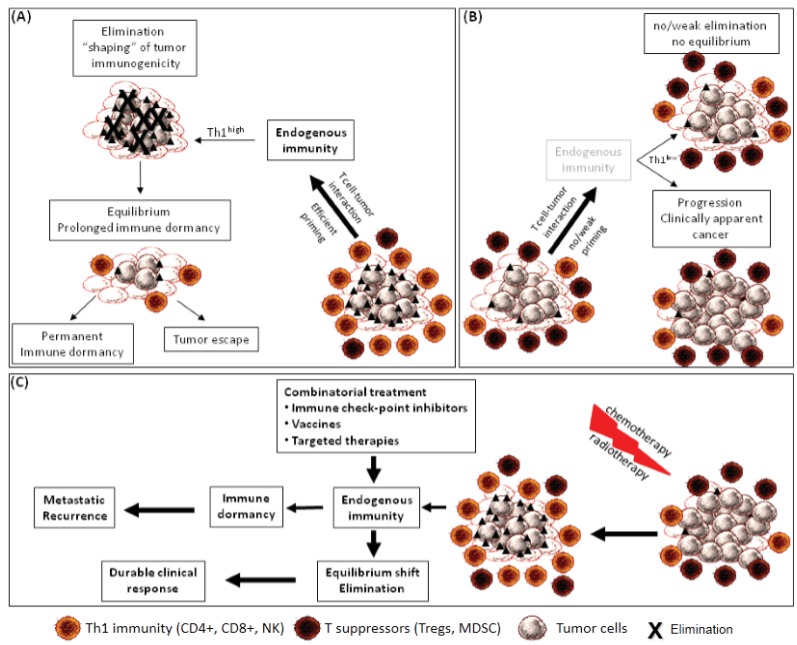
Endogenous immunity possesses a central role to tumor dormancy under an equilibrium condition with immunologic clearance. Endogenous immunity controls immunogenic tumor cells through the processes of elimination and equilibrium. Elimination functions as an extrinsic tumor suppressor in naïve hosts in which innate and adaptive immunity work together to detect and destroy immunogenic tumor cells before they become clinically symptomatic. Certain tumor variants may not be completely eliminated, but their net growth is restricted by immunity control, resulting in an equilibrium state and maintenance of tumor cells in prolonged dormancy. These DTC may induce a host-protective immune response and remain in permanent dormancy. Over a prolonged period, via immune adaptation and genomic instability, DTCs may enter into an attenuated immunogenic status or may acquire a suppressor phenotype and escape from immunesurveillance (**A**). By non-immunogenic tumors endogenous immunity is weak, allowing tumors to grow progressively in an immuno-suppressive tumor microenvironment and give clinically detectable cancers (**B**). Such tumors can be rendered immunogenic via conventional treatments, thus reinstating endogenous antitumor immunity (**C**); Tumor-oriented therapies or immunotherapies alone or combined with standard therapies may potentiate the endogenous immunity to induce durable clinical benefit. Filled triangles show molecules conferring immunogenicity to tumor cells (e.g., MHC, co-stimulatory and adhesion molecules, tum or antigens, DC maturation factors).

The immune system during the elimination phase of immunoediting, through interaction with the autologous tumor cells shapes their immunogenicity and tumorigenicity in a process which practically selects for the tumor variants which will progress later on. We may speculate that a full editing process of the tumor will be completed during the late stages of immune surveillance at which time-period the selective pressure mediated by the immune system has destroyed the immunogenic tumor variants leaving intact, or imposing the development of, less immunogenic ones. In general, the outcome from the equilibrium phase depends on the balance between the strength and duration of the endogenous effector antitumor immunity and tolerance mechanisms developed by the tumor cells [49], making endogenous antitumor immune pathways indispensable for inducing and maintaining tumor dormancy during immune equilibrium. Thus, equilibrium represents a type of tumor dormancy which expands throughout a certain time-frame, during which outgrowth of occult tumors is specifically controlled by the endogenous antitumor immunity. The immune system during the elimination phase of immunoediting, through interaction with the autologous tumor cells shapes their immunogenicity and tumorigenicity in a process which practically selects for the tumor variants which will progress later on. We may speculate that a full editing process of the tumor will be completed during the late stages of immune surveillance at which time-period the selective pressure mediated by the immune system has destroyed the immunogenic tumor variants leaving intact, or imposing the development of, less immunogenic ones. In general, the outcome from the equilibrium phase depends on the balance between the strength and duration of the endogenous effector antitumor immunity and tolerance mechanisms developed by the tumor cells [49], making endogenous antitumor immune pathways indispensable for inducing and maintaining tumor dormancy during immune equilibrium. Thus, equilibrium represents a type of tumor dormancy which expands throughout certain time-frame, during which outgrowth of occult tumors is specifically controlled by the endogenous antitumor immunity (Figure 1A). Tumor immune surveillance is a major component of durable tumor dormancy, provided that tumor cells are capable of sensitizing T cell responses specifically targeting their tumor antigens. This naturally occurring tumor immunogenicity will favor the establishment of tumor dormancy after immune surveillance. Tumor-specific immune memory will then make sure that this immune dormancy will be long-lasting. During equilibrium, tumor cells remain dormant for prolonged periods of time, lasting even for decades, in a process requiring active endogenous immune mechanisms, whereby memory T cells act as the main players inducing and maintaining tumor dormancy via a continuous durable pressure. The indispensable role of endogenous antitumor immunity for sustaining tumor dormancy or even eliminating dormant tumor cells is further supported by accumulating evidence suggesting that metastases may derive from very early disseminated tumor cells (DTC), even before the primary tumor becomes clinically detectable [6]. Such DTC are kept into dormancy or metastatic latency by host-derived CTLs. It will be thus of instrumental importance to identify cellular and/or serum biomarkers which might help to detect dormant disease. In addition, transcriptional profiles from dormant disseminated tumor cells or experimental models of dormancy might help determine whether primary tumors carry a cancer-dormancy “signature”, which might have prognostic and also therapeutic value. However, non-immunogenic or less immunogenic tumors, which are not able to induce tumor-directed effector immunological responses and memory, will proceed relatively fast and will give clinically apparent tumors (Figure 1B) [2]. Such tumors can be rendered immunogenic, for example, following immunogenic chemotherapy or radiotherapy of primary cancers [56,57], and immune-mediated dormancy in this case will keep residual tumor cells under control (Figure 1C). Thus, we may postulate that, during equilibrium, a bidirectional reaction between tumor and immune cells exists, resulting in a mutual modulation, which maintains a balanced status between elimination and dominance of either population. An interruption of this state of immune dormancy might be due to tumor cell escape from immune system control as a result of genomic instability. Specific mutations in such dormant tumor cells may lead to altered expression of tumor antigens, which, once detected, may serve as novel immunotherapeutic targets. In addition, dormant tumor cells may escape from immune control through expression of immune checkpoint ligands directly leading to exhaustion of immune effector T cells. MDSC and Tregs, may also be attracted by the tumor to establish an immunosuppressive tumor microenvironment inducing indirect escape from dormancy [58,59,60,61] (Figure 1C). Thus, the relationship between tumor suppressor status and endogenous adaptive immunity at immune dormancy will be of particular interest in maintaining the equilibrium or even to shift the equilibrium back in favor of immune-mediated elimination of the tumor. Cancer vaccines amplifying antitumor T cell activation while promoting maturation of dendritic cells constitute one promising approach to expand the tumor-specific effector T cell pool. Therapeutic vaccination has also been reported to rescue the dysfunctional endogenous tumor-specific CD8+ T-cell response leading to eradication of long-established tumors [62]. Potentiation of endogenous antitumor immunity by vaccination can be detected through epitope spreading which is defined as the immunity to tumor antigens not included in the vaccine preparation. Such preexisting endogenous antitumor immunity can be boosted during immunizations. Epitope spreading is associated with improved outcomes after the administration of a cancer vaccine [63]. Given the waning immunity followed by late recurrences, it will be imperative to sustain endogenous host-protective immune responses by booster immunizations during active immunotherapies or by strategies reversing tumor-induced immune tolerance, such as targeted therapies with immune checkpoint inhibitors and kinase inhibitors [11] but also via conventional therapies [26], all of which could reinvigorate endogenous antitumor immunity and rise to memory cells.

Interestingly enough, tumor dormancy has also been registered long before immune equilibrium in DTC [64,65] suggesting that tumor dormancy is not exclusively a state of tumor latency that takes place within the primary tumor after tumor cells have been edited, but it may also appear in DTC much earlier before the process of immunoediting. Nonetheless, no matter the stage during which tumor dormancy occurs, it will be important to detect biomarkers for predicting dormant disease. Equilibrium in humans represents the longest phase of the immunoediting process, which, however, is difficult to determine. Consequently, it is practically impossible to detect dormant tumor cells during this phase. DTC from primary tumors may offer a more realistic source to detect dormant tumor cells and to identify dormant gene signatures. Such signatures may additionally improve our understanding of the relationship between immunity and cancer and in this way provide insight into the management of malignant diseases.

## 5. Local and Peripheral Endogenous Immunity in Immune Equilibrium

In their majority, human tumors are infiltrated by elements of the immune system, which establish a dynamic interaction with tumor cells. The result of it is the emergence of tumor variants with altered phenotypes and function, which, in turn, influence the orchestration of their neighboring adaptive immunity. Thus, there is a mutual influence between tumor cells and infiltrating lymphocytes, which establishes an immunological status. The quantity and the quality of this immune contexture at the tumor site have been demonstrated to function as an important prognostic biomarker for colorectal cancer as well as other types of cancer [3,5]. In some solid tumors, high levels of tumor-infiltrating CD4+ T helper (Th), CTLs, and CD45RO+ memory T lymphocytes have been associated with favorable clinical outcomes [66,67]. Consistent with these studies, findings from Wu *et al.* [68] suggested that increased proportions of infiltrating CTLs and other effector immune cells are associated with maintenance of immune-mediated dormancy. Tumor infiltration by CTLs correlates with increased survival in melanoma patients at the early stages of their disease [69]. Moreover, the presence of critical chemokines in a subset of melanoma metastases has been found to enhance the migration of activated CD8+ T cells, which in turn could increase the effectiveness of antitumor immunity and survival [70]. The expression of the T cell activation marker CD69 was also shown to correlate positively with survival and negatively with metastasis in patients with cutaneous melanoma [71]. Conversely, high frequencies of tumor-infiltrating FOXP3+ Tregs often represent a poor prognostic factor [67], although recent studies have questioned this observation by showing that Tregs phenotypically may be misinterpreted and, therefore, in some types of cancer, may associate with favorable prognosis [35]. Cancer treatments may modulate the tumor microenvironment to enhance local adaptive immunity. Therapies designed to target tumor cells have also the potential to induce positive immunomodulatory effects, directly, by acting on effector immune lymphocytes and/or antigen presenting cells, or indirectly, by counteracting the hostile conditions at the tumor site [9,38,42,43]. Such changes may be further potentiated in the setting of active immunotherapies, which will boost the endogenous anticancer immunity. In this scenario, conventional chemotherapy and radiotherapy, but also targeted tumor therapies, will relieve the preexisting anticancer immunity from tumor-induced suppression, which, in the presence of active immunotherapies, will become durable, inducing long-lasting clinical responses. Thus, it will be imperative to sustain such endogenous host-protective immune responses by booster immunizations during active immunotherapies [72,73] or by strategies reversing tumor-induced immune tolerance, such as targeted therapies with immune checkpoint inhibitors and kinase inhibitors [74] but also via conventional therapies [38], all of which could reinvigorate endogenous antitumor immunity and rise to memory cells.

Given that clinically apparent cancers are developed from their dormant ancestors [75], it is conceivable that the prevalence of immune activation *vs.* immune suppression at the tumor site has a strong impact on the maintenance of tumor dormancy during the equilibrium phase. The longevity of tumor dormancy is also important for patients’ overall survival after conventional treatments. Dormant circulating tumor cells and disseminated tumor cells at different organs after chemotherapy provide clinical evidence for treatment-induced tumor dormancy which will be sustainable only if the tumor is immunogenic or has become immunogenic after chemotherapy or radiotherapy [2]. Thus, immune signatures in the periphery, in addition to the intratumoral ones, may also have prognostic significance for patients’ overall survival following standard treatments. The numbers and functions (Th1 *vs.* Th2) of circulating T lymphocytes in cancer patients during and after immunotherapies have been analyzed as potential predictive biomarkers and surrogates for clinical outcome [76]. However, there might be potential drawbacks when trying to establish circulating immune cells as biomarkers. For instance, the frequencies of vaccine-specific T cells in peripheral blood samples during and post active immunotherapies may vary widely when collected at different time-points, hampering to a certain extent the accuracy of assays applied for their detection. This may provide one more obstacle in the field of cancer immunotherapy, in addition to the low frequencies of circulating tumor antigen-specific T cells reported in patients [77]. It should be also considered that depending on the kinetics of their peak response during vaccinations, the vaccine-specific T cells by the time of blood collection may have totally or partially disappeared being sequestered at lymphoid organs or other tissues and therefore will not be detectable in blood samples.

## 6. Immune Editing, Dormancy and Escape from Immune Surveillance

**Figure 2:**
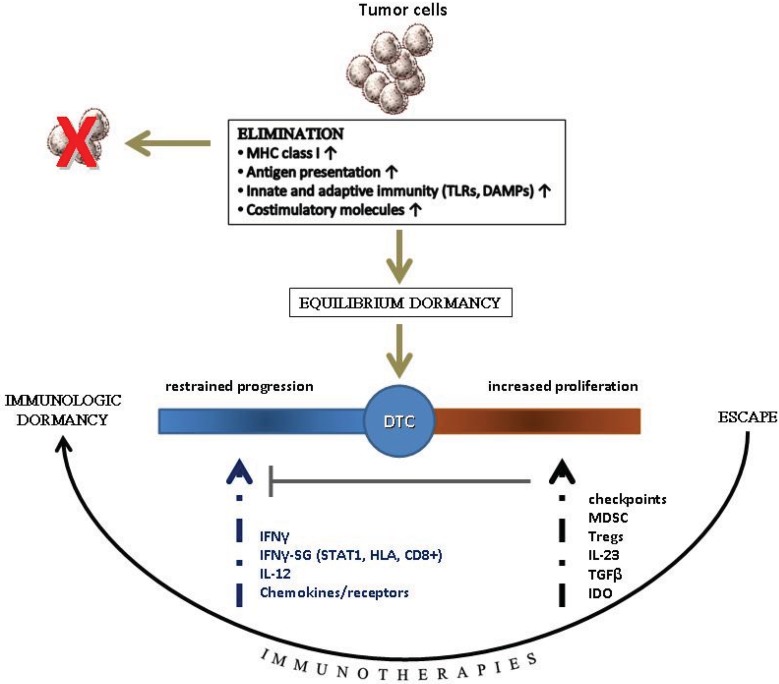
Control of DTC via the immune system: In early tumor development immunogenic tumor clones are recognized and eliminated by both innate and adaptive immune-mediated mechanisms (elimination). In the equilibrium dormancy, the “edited” tumor cells and the adaptive immune system coexist: tumor growth rates are controlled by an active Th1 adaptive immunity. Tumor escape occurs by immunosuppressive cytokines and enzymes, Tregs, MDSCs, and activation of immune checkpoints. Immunotherapies aim to shift the balance from escape to equilibrium dormancy. TLRs: toll-like receptors; DAMPs: danger-associated molecular pattern molecules; IFNγ-SG: IFNγ-stimulated genes; STAT1: signal transducers and activator of transcription 1; TGFβ: transforming growth factor beta; IDO: Indoleamine 2,3-dioxygenase.

There is now accumulating evidence to suggest that in humans there is an equilibrium between tumor dormancy and immune surveillance which emerges after the elimination phase during the process of tumor immunoediting [58]. The most convincing reports on this were those describing the emergence of cancers of donor origin in immunosuppressed transplant recipients [78]. Nevertheless, despite equilibrium, tumors will progress and metastasize at later time-points, suggesting that equilibrium can be disturbed resulting in tumor escape [79]. Immune surveillance controlling tumor dormancy during equilibrium utilizes effector immune pathways similar to those being active during tumor destruction in the elimination phase, mostly including cytotoxic effector/memory T cells, and Th1 cytokines [2]. Tumor dormancy during equilibrium implies that the tumor cells have “survived” the elimination phase but their progression is successfully restrained by immune-mediated mechanisms reflecting activation of Th1-associated factors such as IFN-γ, STAT1, IRF1, IL-12 as well as upregulation of CD8 genes and chemokine receptor pathways (CXCR3/CXCL9–11 and CCR5/CCL3–5) [2,33,65]. An interruption of this equilibrium dormancy imposes the initiation of period during which dormant tumor cells will gain advantages for survival and proliferation via genetic or epigenetic changes, and will dominate and metastasize [80]. It is therefore conceivable that the balance between tumor suppressor status (including suppressor cells and factors) and Th1-associated signature will be of particular interest, in maintaining the equilibrium (Figure 2). Results from studies exploring differences between primary tumors and metastatic lesions showed that a subpopulation of primary tumor cells resembled metastatic tumor cells to an extended degree with respect to gene expression profiles [64]. In a mouse model of melanoma [65], primary tumor cells were found to metastasize during early development of primary tumor formation, before the tumor was clinically detectable. These early metastasized tumor cells were kept dormant by effector CTL for varying periods of time. The results from these studies are suggesting that the metastatic potential of tumor cells is determined early during carcinogenesis and that dormancy also exists in metastasized tumor cells from early primary tumors. Hence, tumor dissemination may occur during, or even before, immunoediting questioning the traditional view that immune surveillance takes place within the developing primary tumor, followed by the dormancy period during which immune “edited” tumor cells are entering the equilibrium phase. Given that metastatic dormancy in disseminated tumor cells is immunologically controlled [2], we may suggest that immune surveillance and equilibrium are processes which may take place autonomously within the primary tumor as well as in the early metastatic lesions. Presently, the immune surveillance mechanisms applied in the primary tumor or the metastatic lesions are not precisely defined and may differ among the various anatomical sites of metastases. Studies from several experimental mouse/tumor models showed that inhibition of T-cell-mediated immunity through T-cell depletion or IFN-γ or IL-12 blocking, induces escape from dormancy [60]. Tumor cells may evade immune surveillance in a process of development of gradual resistance during which the dormant tumor cells are no longer capable of effectively stimulating immune effector mechanisms. Active suppression is an additional mechanism, which may also be used by the tumor cells in this respect. Understanding the mechanisms of immune escape leading to transition from tumor dormancy to tumor domination will surely provide means for novel immunotherapeutics.

## 7. Immune Adaptation

Taking into consideration our present knowledge on cancer progression under the concept of immunoediting, we may conclude that the interaction between the spontaneously arising tumor with the immune system in the surrounding microenvironment must comprise a highly complex network of systems biology which evolves over years of tumor development. The cancer clones that are selected to proceed, manipulate their microenvironment to their own benefit, so that a particular microenvironment supports their growth. According to this scenario, the constant interaction between immune lymphocytes with their neighboring tumor cells will result in mutual phenotype and functional changes, all of which will establish an intratumoral immunologic *status quo*. As we are now having a more clear picture of the mechanisms of tumor escape from immune surveillance, it will be quite important to also find out which mechanisms are being pursued by the immune system in order to overcome this tumor-driven immunologic regime which actually reflects a “contra” editing mediated by the tumor cells, rendering immune lymphocytes at the tumor site dysfunctional. Thus, after the tumor has evaded immune surveillance, either after being “unrecognizable” by immune effectors specifically targeting its tumor antigens or by inactivating these effectors via active suppression, the host’s immune T cell repertoire should be modified in a way to reinvigorate and to attack the tumor by targeting other tumor antigenic peptides. This can be achieved by its capacity to adapt to the tumor-induced immune editing either via active immunotherapies or even spontaneously, in the absence of exogenous immune stimulants. Vaccination of melanoma patients has frequently resulted in tumor regressions, however, not due to an increase in the frequencies of the vaccine-specific T cells, but rather due to an increase in the numbers of T cells targeting tumor antigens other than those included in the vaccine formulation. Thus, activation of patients’ immune system following vaccination may not be solely based on the frequencies of T cells specifically targeted by the vaccine, but rather on the production of functionally active tumor-specific T cells, as a result of a tumor regression process induced by the vaccine, which enables epitope spreading [81]. Such tumor-specific T cell clones may possess functional properties that enable them to migrate to the tumor site, to counterbalance the immunosuppessive milieu within the tumor and to initiate a robust regression process. Thus, even in the event of antigen-loss in tumor variants, these antitumor effectors, targeting a plethora of tumor antigens, will prevent tumor escape, and at the same time will restore any tumor-induced immune compromise. Spontaneous immune adaptation has been documented in one melanoma patient whose immune system, after the tumor initially evaded immune recognition by down regulating the expression of an immunodominant MART-1 peptide, was expanded to generate T cell clones recognizing a tyrosinase subdominant epitope which was presented in the context of tumor’s HLA class I alleles [82]. Such an immunologic response was associated with patients’ survival benefit, suggesting that the adaptive evolution of the endogenous antitumor immunity may overcome barriers set by the tumor. Thus, it is appealing to assume that the ability of the immune system to overcome conditions set via tumor immune editing by generating novel adaptive immunological responses, thereby extending its tumor-reactive T cell repertoire, and exploring the molecular pathways that dictate such responses, may provide efficacious modalities for preventing tumor metastases after immune escape.

## 8. Cancer Stem Cells in Clinical Tumor Dormancy

In the previous paragraphs, we have focused on the role of the immune system, as this is defined from endogenous antitumor responses, in controlling the process of tumor dormancy during the equilibrium phase. However, this may still not provide a complete spectrum of events for the process of tumorigenesis and consequently a better understanding of how tumor cells, by escaping from this immune control, enter their progression cycle and give clinically overt metastases. Thus, by looking the coin from the other side, namely the tumor side, we should realize that it is imperative to understand the sequence of molecular and cellular events which initiate the process of tumor escape from immune surveillance long before the tumor becomes aggressive and progresses to invasive and metastatic processes. Such a knowledge obtained at time periods where the tumor mass is low, will provide the platform for development of more effective therapeutics. From the immunological point of view, these cancer initiating tumor cells in order to be able to avoid immune surveillance, should have no or low immunogenicity. To this point, it is worth mentioning that these cells may gain genetic and epigenetic changes giving rise to immunogenic and fast growing daughter cells, which, however, will be visible and eliminated by immune effector cells [83]. The rest of the cells, being at low frequencies, will belong to a subpopulation of non immunogenic tumor cells, slow growing and incapable of giving metastases [83]. Thus, conceptually, the long-lasting latency periods described for many type of tumors, depends on the presence of a subpopulation of tumor cells which is poorly immunogenic and therefore capable of avoiding immune surveillance mechanisms, but with slow growth rates not able to grow into overt tumors. Consequently, it has been proposed that cancer stem cells (CSCs) meet these criteria, being not only long-lived, but also remarkably well protected from immune-attacks, and largely confined to stem cell niches [83]. Accordingly, latent tumors may be maintained by a niche-constrained reservoir of long-living CSCs that are exempt from immunosurveillance while niche-independent and more immunogenic daughter cells are constantly eliminated. Nevertheless, the fact we have to face is that in some individuals, tumors will override barriers set by the endogenous antitumor immunity and will recur giving clinically detectable metastases. There are two issues which emerge from this pro-tumor situation and require further clarification: first, which changes may have occurred to enable tumors to escape immune surveillance leaving the state of latency and becoming progressively aggressive? And second, how and to which extent tumors are involved in the weakening of the immune system? We may suggest that dormant tumor cells by expressing low but persistent levels of tumor antigens contribute to the maintenance of long-term memory targeting those antigens. On the other hand genetic abnormalities in dormant tumor cells may generate phenotype alterations enabling immune evasion and reactivation of these cells [84]. In a preclinical model, bone-marrow derived indolent tumor cells having down-regulated the expression of certain adhesion and cell lineage markers but displaying increased levels of expression of H-2 class I molecules to present endogenously derived tumor-antigens, could stimulate tumor reactive CD8+ memory T cells directly [85]. In contrast, the absence of adhesion molecules in these tumor cells was related to a strategy of immune evasion. There are also reports suggesting that immune-mediated induction of epigenetic changes in primary tumors leads to tumor antigen loss.

IFN-γ, secreted by TIL promoted CT26 colon carcinoma escape through the down-regulation of the endogenous tumor Ag gp70 [86,87]. In another preclinical model of rat neu-overexpressing mouse mammary carcinoma [88,89], it was demonstrated that that loss of HER-2/neu antigen expression was caused via the induction of epigenetic changes in the presence of IFN-γ producing neu-specific T cell responses. In addition, signals delivered via the IFN-γ receptor resulted in a powerful growth arrest response [90] supporting a model in which IFN-γ produced by activated CD8+ T cells directly mediates growth arrest *in vivo*, thereby maintaining the tumor dormant state. Thus, there must be a balance between tumor antigen-sensitized CTLs and dormant tumor cells which are susceptible to CTL-mediated lysis in the tumor microenvironment. In this scenario, the expression of a certain tumor phenotype (e.g., low levels of adhesion molecule) could complicate CTLs-tumor cell interactions *in situ*, thus allowing for the co-existence of CTLs-sensitive tumor cells and tumor-reactive killers in the tumor microenvironment. It is then easy to imagine a situation where “dormant” tumor cells residing in a metastatic site proliferate at a slow rate and are maintained at a constant population size by the active control of CTLs, which, in turn, are present at elevated frequencies as a result of stimulation by tumor antigens displayed on the surface of dormant tumor cells.

Expansion of CSCs and evasion may include mutations in cell signal transduction pathways known to promote self-renewal of stem-cells and expansion by increasing frequency of cell divisions [91,92,93,94,95,96]. Because it is a long way for such changes, genetic and/or epigenetic, to accumulate, less or non-immunogenic long-lived CSCs represent the best candidates for tumor cell subpopulations to give late recurrences and metastases. Therefore, it is conceivable that enhanced frequencies of cell division in CSCs may associate with a risk for emergence of more aggressive clonal subpopulations. Addressing the second issue, tumors may be passively and actively involved in the weakening of the immune system. Passively, they may be involved by aging, or due to therapy-mediated suppression. Aggressive tumors, however, may actively suppress immune effector cells, and therefore evade immune-surveillance, by secretion of immunosuppressive factors or by recruitment of Tregs and MDSCs, which locally suppress the immune system [97,98,99]. For example, tumors can down-regulate T cell receptor-mediated activation of cytotoxic T lymphocytes, increase anti-apoptotic pathways and induce deficiencies in the antigen processing machinery of antigen-presenting cells [83]. Furthermore, genetic alterations may result in HLA haplotype reduction or loses and deficient expression of tumor antigens that are recognized by immune effectors.

## 9. Conclusions and Future Directions

The relative balance of effector and memory immune cells, on one hand, and immunosuppressive populations in the tumor microenvironment, on the other, determines the fate of the tumor. Altering this balance may affect the immune response and tumor growth, therefore maintaining the equilibrium state. Anticancer therapies including body’s own immune effector arsenal, primarily aim at totally eradicating the evolving cancer so to avoid future recurrences of metastatic disseminated tumor cells. Although this possibility is feasible, nevertheless, the problem we have to face lies to the fact that none of the therapeutic modalities completely eradicates the tumor, leaving behind minimal residual disease (MRD) which is based on the presence of dormant tumor cells disseminated at various anatomical sites. Actually, we do not know much about how this MRD establishes and how long it persists before giving recurrences, however, what we have found out so far is that the immune equilibrium between the endogenous anticancer immune response and the dormant disseminated tumor cells is responsible for keeping the tumor from progressing. Thus, knowledge on the conditions that are necessary for maintaining immune dormancy and inhibiting immune escape may provide the platform for designing more effective strategies for the eradication of disseminated tumor cells. Although much has been proposed in this direction, we believe that the main directions involve immunogenic cell death in residual dormant tumor cells or maintenance of immune dormancy of tumor cells by immune system-oriented and tumor-targeted therapies. We should not neglect that conventional treatments such as chemotherapy and radiotherapy, by exerting anti-proliferative effects and apoptosis, also contribute to the state of cancer dormancy. However, such non-immunogenic dormancy will result in early recurrences after completion of standard treatments. One puzzling question would concern the sequencing of these treatments and the time frame to apply. In fact, standard therapies are mostly inappropriate for long-term maintenance of tumor dormancy, given the fact that they cannot induce tumor-specific memory. More important, these therapies lack specificity, which implies that they are inducing toxic side effects that hamper their long-term application. As a result, malignant cells, which will are rescued from the deleterious effects of cytotoxic treatments, enter a stage of dormancy and will recur and metastasize. Based on this, we conclude it would be preferable if all treatments aiming at eradicating and restraining dormant tumor cells, by potentiating endogenous antitumor immune mechanisms, should be applied in patients in complete remission after surgical excision of their primary tumor, where tumor load is low and thus manageable by the immune system. Second, the identification of biomarker signatures for immune dormancy will be important to apply an additional attack against the tumor after the failure of primary treatments. Finally, another important aspect will be the improved understanding of mechanisms leading to tumor dormancy, which will also uncover novel therapeutic options, thus preventing tumor recurrences and metastases.

## References

[1] Shankaran V., Ikeda H., Bruce A.T., White J.M., Swanson P.E., Old L.J., Schreiber R.D. (2001). IFNgamma and lymphocytes prevent primary tumour development and shape tumour immunogenicity. Nature.

[2] Manjili M.H. (2014). The inherent premise of immunotherapy for cancer dormancy. Cancer Res..

[3] Quezada S.A., Peggs K.S., Simpson T.R., Allison J.P. (2011). Shifting the equilibrium in cancer immunoediting: From tumor tolerance to eradication. Immunol. Rev..

[4] Bindea G., Mlecnik B., Fridman W.H., Pages F., Galon J. (2010). Natural immunity to cancer in humans. Curr. Opin. Immunol..

[5] Fridman W.H., Pages F., Sautes-Fridman C., Galon J. (2012). The immune contexture in human tumours: Impact on clinical outcome. Nature Rev. Cancer.

[6] Schlom J., Hodge J.W., Palena C., Tsang K.Y., Jochems C., Greiner J.W., Farsaci B., Madan R.A., Heery C.R., Gulley J.L. (2014). Therapeutic cancer vaccines. Adv. Cancer Res..

[7] Melero I., Gaudernack G., Gerritsen W., Huber C., Parmiani G., Scholl S., Thatcher N., Wagstaff J., Zielinski C., Faulkner I. (2014). Therapeutic vaccines for cancer: An overview of clinical trials. Nature Rev. Clin. Oncol..

[8] Vanneman M., Dranoff G. (2012). Combining immunotherapy and targeted therapies in cancer treatment. Nat. Rev. Cancer.

[9] Gajewski T.F., Woo S.R., Zha Y., Spaapen R., Zheng Y., Corrales L., Spranger S. (2013). Cancer immunotherapy strategies based on overcoming barriers within the tumor microenvironment. Curr. Opin. Immunol..

[10] Galluzzi L., Vacchelli E., Eggermont A., Fridman W.H., Galon J., Sautes-Fridman C., Tartour E., Zitvogel L., Kroemer G. (2012). Trial watch: Adoptive cell transfer immunotherapy. Oncoimmunology.

[11] Yamada A., Sasada T., Noguchi M., Itoh K. (2013). Next-generation peptide vaccines for advanced cancer. Cancer Sci..

[12] Kantoff P.W., Higano C.S., Shore N.D., Berger E.R., Small E.J., Penson D.F., Redfern C.H., Ferrari A.C., Dreicer R., Sims R.B. (2010). Sipuleucel-T immunotherapy for castration-resistant prostate cancer. N. Engl. J. Med..

[13] Gulley J.L., Giacchino J.L., Breitmeyer J.B., Franzusoff A.J., Panicali D., Schlom J., Kantoff P.W. (2015). Prospect: A randomized double-blind phase 3 efficacy study of PROSTVAC-VF immunotherapy in men with asymptomatic/minimally symptomatic metastatic castration-resistant prostate cancer. J. Clin. Oncol..

[14] Morse M.A., Niedzwiecki D., Marshall J.L., Garrett C., Chang D.Z., Aklilu M., Crocenzi T.S., Cole D.J., Dessureault S., Hobeika A.C. (2013). A randomized phase II study of immunization with dendritic cells modified with poxvectors encoding CEA and MUC1 compared with the same poxvectors plus GM-CSF for resected metastatic colorectal cancer. Ann. Surg..

[15] Emens L.A., Asquith J.M., Leatherman J.M., Kobrin B.J., Petrik S., Laiko M., Levi J., Daphtary M.M., Biedrzycki B., Wolff A.C. (2009). Timed sequential treatment with cyclophosphamide, doxorubicin, and an allogeneic granulocyte-macrophage colony-stimulating factor-secreting breast tumor vaccine: A chemotherapy dose-ranging factorial study of safety and immune activation. J. Clin. Oncol..

[16] Kruit W.H., Suciu S., Dreno B., Mortier L., Robert C., Chiarion-Sileni V., Maio M., Testori A., Dorval T., Grob J.J. (2013). Selection of immunostimulant AS15 for active immunization with MAGE-A3 protein: Results of a randomized phase II study of the European Organisation for Research and Treatment of Cancer Melanoma Group in Metastatic Melanoma. J. Clin. Oncol..

[17] Vansteenkiste J., Zielinski M., Linder A., Dahabreh J., Gonzalez E.E., Malinowski W., Lopez-Brea M., Vanakesa T., Jassem J., Kalofonos H. (2013). Adjuvant MAGE-A3 immunotherapy in resected non-small-cell lung cancer: Phase II randomized study results. J. Clin. Oncol..

[18] Rini B.I., Eisen T., Stenzl A., Brugger W., Weinschenk T., Mahr A., Fritsche J., Hilf N., Mendrzyk R., Lindner J. (2015). IMA901 multipeptide vaccine randomized international PHASE III trial (IMPRINT): A randomized, controlled study investigating IMA901 multipeptide cancer vaccine in patients receiving sunitinib as first-line therapy for advanced/metastatic RCC. J. Clin. Oncol..

[19] Schuster S.J., Neelapu S.S., Gause B.L., Janik J.E., Muggia F.M., Gockerman J.P., Winter J.N., Flowers C.R., Nikcevich D.A., Sotomayor E.M. (2011). Vaccination with patient-specific tumor-derived antigen in first remission improves disease-free survival in follicular lymphoma. J. Clin. Oncol..

[20] Kyte J.A., Gaudernack G., Dueland S., Trachsel S., Julsrud L., Aamdal S. (2011). Telomerase peptide vaccination combined with temozolomide: A clinical trial in stage iv melanoma patients. Clin. Cancer Res..

[21] Dreicer R., Stadler W.M., Ahmann F.R., Whiteside T., Bizouarne N., Acres B., Limacher J.M., Squiban P., Pantuck A. (2009). MVA-MUC1-IL2 vaccine immunotherapy (TG4010) improves PSA doubling time in patients with prostate cancer with biochemical failure. Investig. New Drugs.

[22] Ramlau R., Quoix E., Rolski J., Pless M., Lena H., Levy E., Krzakowski M., Hess D., Tartour E., Chenard M.P. (2008). A phase II study OF TG4010 (MVA-MUC1-IL2) in association with chemotherapy in patients with stage III/IV non-small cell lung cancer. J. Thorac. Oncol..

[23] Quoix E., Ramlau R., Westeel V., Papai Z., Madroszyk A., Riviere A., Koralewski P., Breton J.L., Stoelben E., Braun D. (2011). Therapeutic vaccination with TG4010 and first-line chemotherapy in advanced non-small-cell lung cancer: A controlled phase 2B trial. Lancet. Oncol..

[24] Sampson J.H., Heimberger A.B., Archer G.E., Aldape K.D., Friedman A.H., Friedman H.S., Gilbert M.R., Herndon J.E., McLendon R.E., Mitchell D.A. (2010). Immunologic escape after prolonged progression-free survival with epidermal growth factor receptor variant III peptide vaccination in patients with newly diagnosed glioblastoma. J. Clin. Oncol..

[25] Butts C., Murray N., Maksymiuk A., Goss G., Marshall E., Soulieres D., Cormier Y., Ellis P., Price A., Sawhney R. (2005). Randomized phase IIB trial of BLP25 liposome vaccine in stage IIIB and IV non-small-cell lung cancer. J. Clin. Oncol..

[26] Melero I., Grimaldi A.M., Perez-Gracia J.L., Ascierto P.A. (2013). Clinical development of immunostimulatory monoclonal antibodies and opportunities for combination. Clin. Cancer Res..

[27] Jochems C., Tucker J.A., Tsang K.Y., Madan R.A., Dahut W.L., Liewehr D.J., Steinberg S.M., Gulley J.L., Schlom J. (2014). A combination trial of vaccine plus ipilimumab in metastatic castration-resistant prostate cancer patients: Immune correlates. Cancer Immunol. Immunother..

[28] Madan R.A., Mohebtash M., Arlen P.M., Vergati M., Rauckhorst M., Steinberg S.M., Tsang K.Y., Poole D.J., Parnes H.L., Wright J.J. (2012). Ipilimumab and a poxviral vaccine targeting prostate-specific antigen in metastatic castration-resistant prostate cancer: A phase 1 dose-escalation trial. Lancet. Oncol..

[29] Dranoff G. (2013). Immunotherapy at large: Balancing tumor immunity and inflammatory pathology. Nat. Med..

[30] Kwek S.S., Cha E., Fong L. (2012). Unmasking the immune recognition of prostate cancer with CTLA4 blockade. Nat. Rev. Cancer.

[31] Luke J.J., Ott P.A. (2013). Kinase inhibitors and immune check-point blockade for the treatment of metastatic melanoma and advanced cancer: Synergistic or antagonistic?. Expert Opin. Pharmacother..

[32] Peng W., Lizee G., Hwu P. (2013). Blockade of the PD-1 pathway enhances the efficacy of adoptive cell therapy against cancer. Oncoimmunology.

[33] Galon J., Angell H.K., Bedognetti D., Marincola F.M. (2013). The continuum of cancer immunosurveillance: Prognostic, predictive, and mechanistic signatures. Immunity.

[34] Galon J., Mlecnik B., Bindea G., Angell H.K., Berger A., Lagorce C., Lugli A., Zlobec I., Hartmann A., Bifulco C. (2014). Towards the introduction of the “immunoscore” in the classification of malignant tumours. J. Pathol..

[35] Baxevanis C.N., Papamichail M., Perez S.A. (2013). Immune classification of colorectal cancer patients: Impressive but how complete?. Expert Opin. Pharmacother..

[36] Denkert C., von Minckwitz G., Brase J.C., Sinn B.V., Gade S., Kronenwett R., Pfitzner B.M., Salat C., Loi S., Schmitt W.D. (2015). Tumor-infiltrating lymphocytes and response to neoadjuvant chemotherapy with or without carboplatin in human epidermal growth factor receptor 2-positive and triple-negative primary breast cancers. J. Clin. Oncol..

[37] Matsumoto H., Koo S.L., Dent R., Tan P.H., Iqbal J. (2015). Role of inflammatory infiltrates in triple negative breast cancer. J. Clin. Pathol..

[38] Zitvogel L., Kepp O., Kroemer G. (2011). Immune parameters affecting the efficacy of chemotherapeutic regimens. Nat. Rev. Clin. Oncol..

[39] Zitvogel L., Galluzzi L., Smyth M.J., Kroemer G. (2013). Mechanism of action of conventional and targeted anticancer therapies: Reinstating immunosurveillance. Immunity.

[40] Antonarakis E.S., Drake C.G. (2012). Combining immunological and androgen-directed approaches: An emerging concept in prostate cancer immunotherapy. Curr. Opin. Oncol..

[41] Formenti S.C., Demaria S. (2013). Combining radiotherapy and cancer immunotherapy: A paradigm shift. J. Nal. Cancer Inst..

[42] Kalbasi A., June C.H., Haas N., Vapiwala N. (2013). Radiation and immunotherapy: A synergistic combination. J. Clin. Investig..

[43] Ribas A., Wolchok J.D. (2013). Combining cancer immunotherapy and targeted therapy. Curr. Opin. Immunol..

[44] Topalian S.L., Drake C.G., Pardoll D.M. (2015). Immune checkpoint blockade: A common denominator approach to cancer therapy. Cancer Cell.

[45] Rizvi N.A., Hellmann M.D., Snyder A., Kvistborg P., Makarov V., Havel J.J., Lee W., Yuan J., Wong P., Ho T.S. (2015). Cancer immunology. Mutational landscape determines sensitivity to PD-1 blockade in non-small cell lung cancer. Science.

[46] Schumacher T.N., Schreiber R.D. (2015). Neoantigens in cancer immunotherapy. Science.

[47] Vesely M.D., Kershaw M.H., Schreiber R.D., Smyth M.J. (2011). Natural innate and adaptive immunity to cancer. Ann. Rev. Immunol..

[48] Dunn G.P., Bruce A.T., Ikeda H., Old L.J., Schreiber R.D. (2002). Cancer immunoediting: From immunosurveillance to tumor escape. Nat. Immunol..

[49] Schreiber R.D., Old L.J., Smyth M.J. (2011). Cancer immunoediting: Integrating immunity’s roles in cancer suppression and promotion. Science.

[50] Garrido F., Cabrera T., Lopez-Nevot M.A., Ruiz-Cabello F. (1995). HLA class I antigens in human tumors. Adv. Cancer Res..

[51] Seliger B., Ritz U., Abele R., Bock M., Tampe R., Sutter G., Drexler I., Huber C., Ferrone S. (2001). Immune escape of melanoma: First evidence of structural alterations in two distinct components of the mhc class I antigen processing pathway. Cancer Res..

[52] Matsushita H., Vesely M.D., Koboldt D.C., Rickert C.G., Uppaluri R., Magrini V.J., Arthur C.D., White J.M., Chen Y.S., Shea L.K. (2012). Cancer exome analysis reveals a T-cell-dependent mechanism of cancer immunoediting. Nature.

[53] Romero I., Garrido C., Algarra I., Collado A., Garrido F., Garcia-Lora A.M. (2014). T lymphocytes restrain spontaneous metastases in permanent dormancy. Cancer Res..

[54] Garrido C., Paco L., Romero I., Berruguilla E., Stefansky J., Collado A., Algarra I., Garrido F., Garcia-Lora A.M. (2012). MHC class I molecules act as tumor suppressor genes regulating the cell cycle gene expression, invasion and intrinsic tumorigenicity of melanoma cells. Carcinogenesis.

[55] Romero I., Garrido F., Garcia-Lora A.M. (2014). Metastases in immune-mediated dormancy: A new opportunity for targeting cancer. Cancer Res..

[56] Dewan M.Z., Galloway A.E., Kawashima N., Dewyngaert J.K., Babb J.S., Formenti S.C., Demaria S. (2009). Fractionated but not single-dose radiotherapy induces an immune-mediated abscopal effect when combined with anti-CTLA-4 antibody. Clin. Cancer Res..

[57] Vacchelli E., Aranda F., Eggermont A., Galon J., Sautes-Fridman C., Cremer I., Zitvogel L., Kroemer G., Galluzzi L. (2014). Trial watch: Chemotherapy with immunogenic cell death inducers. Oncoimmunology.

[58] Hensel J.A., Flaig T.W., Theodorescu D. (2013). Clinical opportunities and challenges in targeting tumour dormancy. Nat. Rev. Clin. Oncol..

[59] Koebel C.M., Vermi W., Swann J.B., Zerafa N., Rodig S.J., Old L.J., Smyth M.J., Schreiber R.D. (2007). Adaptive immunity maintains occult cancer in an equilibrium state. Nature.

[60] Quesnel B. (2008). Tumor dormancy and immunoescape. APMIS.

[61] Teng M.W., Vesely M.D., Duret H., McLaughlin N., Towne J.E., Schreiber R.D., Smyth M.J. (2012). Opposing roles for IL-23 and IL-12 in maintaining occult cancer in an equilibrium state. Cancer Res..

[62] Binder D.C., Engels B., Arina A., Yu P., Slauch J.M., Fu Y.X., Karrison T., Burnette B., Idel C., Zhao M. (2013). Antigen-specific bacterial vaccine combined with anti-PD-l1 rescues dysfunctional endogenous t cells to reject long-established cancer. Cancer Immunol. Res..

[63] Disis M.L. (2011). Immunologic biomarkers as correlates of clinical response to cancer immunotherapy. Cancer Immunol. Immunother..

[64] Bernards R., Weinberg R.A. (2002). A progression puzzle. Nature.

[65] Eyles J., Puaux A.L., Wang X., Toh B., Prakash C., Hong M., Tan T.G., Zheng L., Ong L.C., Jin Y. (2010). Tumor cells disseminate early, but immunosurveillance limits metastatic outgrowth, in a mouse model of melanoma. J. Clin. Investig..

[66] Pages F., Kirilovsky A., Mlecnik B., Asslaber M., Tosolini M., Bindea G., Lagorce C., Wind P., Marliot F., Bruneval P. (2009). *In situ* cytotoxic and memory T cells predict outcome in patients with early-stage colorectal cancer. J. Clinical Oncol..

[67] Whiteside T.L. (2014). Regulatory T cell subsets in human cancer: Are they regulating for or against tumor progression?. Cancer Immunol. Immunother..

[68] Wu X., Peng M., Huang B., Zhang H., Wang H., Xue Z., Zhang L., Da Y., Yang D., Yao Z. (2013). Immune microenvironment profiles of tumor immune equilibrium and immune escape states of mouse sarcoma. Cancer Lett..

[69] Piras F., Colombari R., Minerba L., Murtas D., Floris C., Maxia C., Corbu A., Perra M.T., Sirigu P. (2005). The predictive value of CD8, CD4, CD68, and human leukocyte antigen-D-related cells in the prognosis of cutaneous malignant melanoma with vertical growth phase. Cancer.

[70] Harlin H., Meng Y., Peterson A.C., Zha Y., Tretiakova M., Slingluff C., McKee M., Gajewski T.F. (2009). Chemokine expression in melanoma metastases associated with CD8+ T-cell recruitment. Cancer Res..

[71] Hillen F., Baeten C.I., van de Winkel A., Creytens D., van der Schaft D.W., Winnepenninckx V., Griffioen A.W. (2008). Leukocyte infiltration and tumor cell plasticity are parameters of aggressiveness in primary cutaneous melanoma. Cancer Immunol. Immunother. CII.

[72] Perez S.A., Anastasopoulou E.A., Papamichail M., Baxevanis C.N. (2014). AE37 peptide vaccination in prostate cancer: Identification of biomarkers in the context of prognosis and prediction. Cancer Immunol. Immunother..

[73] Perez S.A., Anastasopoulou E.A., Tzonis P., Gouttefangeas C., Kalbacher H., Papamichail M., Baxevanis C.N. (2013). AE37 peptide vaccination in prostate cancer: A 4-year immunological assessment updates on a phase I trial. Cancer Immunol. Immunother..

[74] Palena C., Schlom J. (2010). Vaccines against human carcinomas: Strategies to improve antitumor immune responses. J. Biomed. Biotechnol..

[75] Aguirre-Ghiso J.A. (2007). Models, mechanisms and clinical evidence for cancer dormancy. Nat. Rev. Cancer.

[76] Whiteside T.L. (2013). Immune responses to cancer: Are they potential biomarkers of prognosis?. Front. Oncol..

[77] Russo V., Pilla L., Lunghi F., Crocchiolo R., Greco R., Ciceri F., Maggioni D., Fontana R., Mukenge S., Rivoltini L. (2013). Clinical and immunologic responses in melanoma patients vaccinated with MAGE-A3-genetically modified lymphocytes. Int. J. Cancer.

[78] Andres A. (2005). Cancer incidence after immunosuppressive treatment following kidney transplantation. Crit. Rev. Oncol. Hematol..

[79] Patel P., Chen E.I. (2012). Cancer stem cells, tumor dormancy, and metastasis. Front. Endocrinol..

[80] Kitamura T., Qian B.Z., Pollard J.W. (2015). Immune cell promotion of metastasis. Nat. Rev. Immunol..

[81] Ikeda H., Lethe B., Lehmann F., van Baren N., Baurain J.F., de Smet C., Chambost H., Vitale M., Moretta A., Boon T. (1997). Characterization of an antigen that is recognized on a melanoma showing partial HLA loss by CTL expressing an NK inhibitory receptor. Immunity.

[82] Yamshchikov G.V., Mullins D.W., Chang C.C., Ogino T., Thompson L., Presley J., Galavotti H., Aquila W., Deacon D., Ross W. (2005). Sequential immune escape and shifting of T cell responses in a long-term survivor of melanoma. J. Immunol..

[83] Bruttel V.S., Wischhusen J. (2014). Cancer stem cell immunology: Key to understanding tumorigenesis and tumor immune escape?. Front. Immunol..

[84] Kim R.S., Avivar-Valderas A., Estrada Y., Bragado P., Sosa M.S., Aguirre-Ghiso J.A., Segall J.E. (2012). Dormancy signatures and metastasis in estrogen receptor positive and negative breast cancer. PLoS ONE.

[85] Mahnke Y.D., Schwendemann J., Beckhove P., Schirrmacher V. (2005). Maintenance of long-term tumour-specific T-cell memory by residual dormant tumour cells. Immunology.

[86] Beatty G.L., Paterson Y. (2000). IFN-gamma can promote tumor evasion of the immune system *in vivo* by down-regulating cellular levels of an endogenous tumor antigen. J. Immunol..

[87] Beatty G.L., Paterson Y. (2001). Regulation of tumor growth by IFN-gamma in cancer immunotherapy. Immunol. Res..

[88] Kmieciak M., Knutson K.L., Dumur C.I., Manjili M.H. (2007). Her-2/neu antigen loss and relapse of mammary carcinoma are actively induced by T cell-mediated anti-tumor immune responses. Eur. J. Immunol..

[89] Kmieciak M., Payne K.K., Wang X.Y., Manjili M.H. (2013). IFN-gamma ralpha is a key determinant of CD8+ T cell-mediated tumor elimination or tumor escape and relapse in FVB mouse. PLoS ONE.

[90] Farrar J.D., Katz K.H., Windsor J., Thrush G., Scheuermann R.H., Uhr J.W., Street N.E. (1999). Cancer dormancy. VII. A regulatory role for CD8+ T cells and IFN-gamma in establishing and maintaining the tumor-dormant state. J. Immunol..

[91] Campbell C., Risueno R.M., Salati S., Guezguez B., Bhatia M. (2008). Signal control of hematopoietic stem cell fate: Wnt, notch, and hedgehog as the usual suspects. Curr. Opin. Hematol..

[92] Li Q., Bohin N., Wen T., Ng V., Magee J., Chen S.C., Shannon K., Morrison S.J. (2013). Oncogenic nras has bimodal effects on stem cells that sustainably increase competitiveness. Nature.

[93] Lu C., Ward P.S., Kapoor G.S., Rohle D., Turcan S., Abdel-Wahab O., Edwards C.R., Khanin R., Figueroa M.E., Melnick A. (2012). IDH mutation impairs histone demethylation and results in a block to cell differentiation. Nature.

[94] Taipale J., Beachy P.A. (2001). The hedgehog and wnt signalling pathways in cancer. Nature.

[95] Takebe N., Harris P.J., Warren R.Q., Ivy S.P. (2011). Targeting cancer stem cells by inhibiting Wnt, Notch, and Hedgehog pathways. Nat. Rev. Clin. Oncol..

[96] Zardawi S.J., O’Toole S.A., Sutherland R.L., Musgrove E.A. (2009). Dysregulation of Hedgehog, Wnt and Notch signalling pathways in breast cancer. Histol. Histopathol..

[97] Kaplan R.N., Riba R.D., Zacharoulis S., Bramley A.H., Vincent L., Costa C., MacDonald D.D., Jin D.K., Shido K., Kerns S.A. (2005). VEGFR1-positive haematopoietic bone marrow progenitors initiate the pre-metastatic niche. Nature.

[98] Sceneay J., Smyth M.J., Moller A. (2013). The pre-metastatic niche: Finding common ground. Cancer Metastasis Rev..

[99] Zitvogel L., Tesniere A., Kroemer G. (2006). Cancer despite immunosurveillance: Immunoselection and immunosubversion. Nat. Rev. Immunol..

